# MiR-143 Regulates Milk Fat Synthesis by Targeting Smad3 in Bovine Mammary Epithelial Cells

**DOI:** 10.3390/ani10091453

**Published:** 2020-08-19

**Authors:** Li Zhang, Zhang-Qing Wu, Yu-Juan Wang, Meng Wang, Wu-Cai Yang

**Affiliations:** College of Animal Science and Technology, Northwest A&F University, Xianyang 712100 Shaanxi, China; frankfrank4@163.com (L.Z.); w2452711225@126.com (Z.-Q.W.); 18792683933@163.com (Y.-J.W.); wangmeng1001@nwafu.edu.cn (M.W.)

**Keywords:** mammary epithelial cells, miR-143, triglyceride, Smad3

## Abstract

**Simple Summary:**

The fat content of milk is the main determinant of its nutritional value, and therefore the study of milk fat synthesis has often received a special focus in research. MicroRNAs (miRNAs) are short RNA sequences which have a crucial function in the synthesis of milk fat. miR-143 is one of the miRNAs closely related to lipid metabolism. In this study, the results showed that miR-143 significantly promoted lipid droplet formation and increased the level of intracellular triglyceride (TAG) via increasing the lipid synthesis related genes such as PPARγ, FASN, SCD1, CEBPβ, and SREBP1 by targeting Smad3. Herein, we constructed a miR-143-Smad3 regulatory network map, which revealed the interactions between miR-143 and Smad3 in milk fat synthesis. These findings provide an insight into understanding the theoretical basis of the genes and can thus be applied in the molecular breeding of dairy cows.

**Abstract:**

Milk fat is the main nutritional component of milk and is also an important indicator for evaluating milk quality. Substantial evidence has implicated miRNAs in the synthesis of milk fat. miR-143 is one of the miRNAs closely related to lipid metabolism. Herein, miR-143 upregulation remarkably promoted the production of lipid droplets and increased the level of intracellular triglyceride (TAG). Meanwhile, miR-143 suppression overtly repressed TAG synthesis and lipid droplet accumulation in bovine mammary epithelial cells (BMECs). At the same time, miR-143 significantly upregulated the genes associated with lipid synthesis, including PPARγ, SCD1, CEBPβ, and SREBP1. To examine the regulatory mechanism of miR-143 in milk fat synthesis, Smad3 was predicted as a new potential miR-143 target gene by TargetScan. Further studies found that miR-143 expression was inversely related to the levels of Smad3 mRNA and protein. Furthermore, luciferase reporter assays confirmed Smad3 to be a miR-143 direct target. Moreover, Smad3 gene silencing significantly increased intracellular TAG level in BMECs. These findings revealed that miR-143 promotes the TAG synthesis in BMECs via increasing the lipid synthesis related gens expression by targeting Smad3. The results of this study can be exploited in devising novel approaches for improving the nutritional value of milk in dairy cows.

## 1. Introduction

Cow milk is a natural food with high nutritional benefits and is therefore considered as basic food in many diets. Milk fat is the main determinant of its nutritional value, and as such it is usually applied to indicate milk quality [1,2], and therefore its synthesis process has been widely studied [3,4,5]. Milk fat synthesis is influenced by several factors, such as heredity, hormone, physiology, environment, and nutrition [6,7,8], but the understanding of the regulation mechanism of milk fat synthesis is still minimal.

MicroRNAs (miRNAs) are short RNA sequences that inhibit mRNA translation and protein synthesis by binding to a mRNA of the target [9]. Recent evidence shows that miRNAs play a crucial function in different biological processes, including cell differentiation, migration, proliferation, apoptosis, and survival [10]. Furthermore, miRNAs play an essential function in the synthesis of milk fat [8]. Shen et al. [6] identified 97 differential expressed miRNAs in the mammary epithelial cells of high-fat and low-fat dairy cows. Further studies found that miR-103, miR-17-5p, and miR-148a enhanced milk fat synthesis by facilitating lipid droplet formation and accumulation of triglycerides [11,12], while miR-181a, mir-34b, and mir-130a inhibit the synthesis of milk fat by downregulating the expression of key genes of lipid synthesis [8,13].

miR-143 is one of the miRNAs closely linked to lipid metabolism [14], which regulates adipocyte differentiation and triglycerides synthesis by modulating the expression of target genes [15]. Levels of miR-143 have been shown to be higher in differentiating adipocytes. Moreover, miR-143 inhibition successfully suppresses adipocyte differentiation [16]. Meantime, miR-143 overexpression promotes fat formation via accelerating lipid droplet aggregation and inhibiting the synthesis of glycerol and free fatty acids in adipocytes [17,18]. Furthermore, Shen et al. [6] found that miR-143 expression in mammary epithelial cells of the high milk fat group was overtly higher relative to that of the low milk fat group [6]. Given this, we speculated that miR-143 might participate in the milk fat synthesis process in bovine mammary epithelial cells (BMECs).

## 2. Materials and Methods

### 2.1. Cell Culture and Transfection

BMECs were extracted from mammary gland tissue of Holstein cows in mid lactation following published protocols [4]. The BMECs were cultured in basal DMEM/F12 medium comprising fetal bovine serum (10%), 100 units/mL penicillin, 100 µg/mL streptomycin, 5 µg/mL bovine insulin, and 1 µg/mL hydrocortisone in 3761 °C in a humidified atmosphere with 5% CO_2_. For lactogenesis induction, BMECs were grown for 48 h in an induction medium composed of basal medium supplemented with 2 µg/mL bovine prolactin (Millipore, Sigma) before conducting the initial experiments. Subsequently, some cells were transfected with a miR-143 mimic (50 nM), a miR-143 inhibitor (200 nM), si-Smad3 (100nM), and the corresponding negative controls (Songon Biotech Co., Ltd., Shanghai, China) as per the manufacturer’s protocol. The sequences for si-Smad3 is as follows: Sense 5′-GCGUGAAUCCCUACCACUATT-3′ and antisense 5′-UAGUGGUAGG GAUUCACGCTT-3′. Unless otherwise mentioned, all the reagents used in this stage were purchased from ThermoFisher Scientific, Shanghai, China.

### 2.2. Gene Expression Assay

TRIzol reagent (Sigma, Louis, MO, USA) was utilized in the isolation of total RNA as per the instructions of the manufacturer. The quality of total RNA was checked via a NanoDrop 1000 (ThermoFisher scientific, Shanghai, China). The OD260/280 value for all RNA samples was from 1.9 to 2.0, and the OD260/230 value was approximately 2.0. To synthesize cDNA, we employed a high-capacity cDNA reverse transcription kit (Takara, Dalian, China) for mRNA expression assay and PrimeScript miRNA RT-PCR kit (Tiangen, Beijing, China) for analyzing miRNA expression. The internal control used to assess mRNA expression was the UXT gene [19], whereas, for miRNA expression, we utilized the U6 gene [20]. Real-time PCR primers (Table 1) were designed and synthesized by TSINGKE Biological Technology (Xi’an, China). The qRT-PCR was carried out using SYBR Premix Ex Taq II (Takara, Dalian, China) together with miRcute miRNA qPCR Detection Kit (Tiangen, Beijing, China). The reaction was carried out on a 7500 Real-Time PCR system (Applied Biosystems Inc., Foster City, CA, USA). The 2−ΔΔCt method was applied in the calculation of relative gene expression [21].

### 2.3. Oil Red O Staining and Triglyceride Assay

BMECs were rinsed thrice in phosphate buffered saline (PBS, including KCl 0.02%, NaCl 0.8%, KH_2_PO_4_ 0.02% and Na_2_HPO_4_ 0.29% in deionized distill water), then fixed for 40 min in 10% paraformaldehyde (1ml PBS, 0.5 µL 10N NaOH, and 0.1 g paraformaldehyde) at 4 °C. After washing in PBS, the cells were stained with 5% Oil Red O in isopropanol for 40 min. Next, the cells were cleaned by PBS, and the lipid droplets were microscopically examined. A cell/tissue triglyceride assay kit (Applygen Technologies, Beijing, China) was used to measure the amount of intracellular TAG, as per the manufacturer’s recommended protocol.

### 2.4. Luciferase Reporter Assay

MiR-143 target genes and miRNA binding sites were identified using TargetScan [22]. The mature sequence of miR-143 was obtained from the miRBase Sequence Database (miRDB, http://www.mirbase.org). Reporter constructs for luciferase were generated through the synthesis of 3′-UTR wild-type (WT) and mutation type (MUT) of Smad3 sequences by ShengGong (Songon Biotech Co., Ltd., Shanghai, China) and cloned into the psiCHECK-2 vector (Promega, Madison, WI, USA) at the NotI and XhoI restriction sites (Promega, Madison, WI, USA). BMECs were grown in 12-well plates and transfected at 70% confluence using Lipofectamine 3000 reagent (Invitrogen, Waltham, MA, USA). About 0.16 µg of MUT vector/Smad-3′ UTR WT was co-transfected with 5 pmol miR-143 mimics/a negative control into BMECs. Luciferase reporter assay system (Promega, Madison, WI, USA) was utilized to assess the luciferase activity following 48 h of transfection.

### 2.5. Western Blot Assay

BMECs were collected using 0.25% trypsin (Solarbio, Beijing, China) and lysed in RIPA buffer (Solarbio, Beijing, China) containing 1% PMSF (Pierce, Rockford, IL, USA). Western blot was carried out as described previously [5]. Primary antibodies against β-actin (mAbcam 8226) (1:2000, Abcam), Smad3 (C679H) (1:1000, Cell Signaling Technology), and HRP goat Anti-Rabbit IgG secondary antibody (ab97051) (1:2000, Abcam) were used. Chemiluminescent ECL Western blot system (Pierce, Rockford, IL, USA) was utilized in signal detection.

### 2.6. Statistical Analysis

The analyses were completed using GraphPad Prism 6.01 software. Data were presented as the mean ± SD. Each experiment contained at least three replicates. Student’s *t*-test was used for pairwise comparisons. Statistical significance was detected at *p* < 0.05, *p* < 0.01, and *p* < 0.001.

## 3. Results

### 3.1. MiR-143 Promotes Triglyceride and Lipid Droplet Accumulation in BMECs

**Figure 1:**
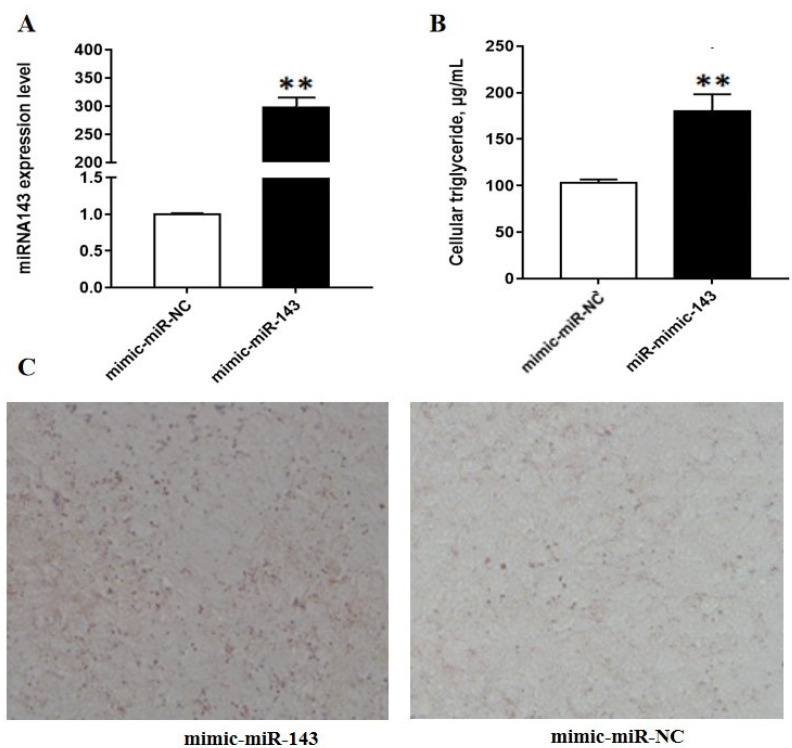
Effects of miR-143 mimic on milk fat synthesis. (**A**) miR-143 expression following miR-143 mimic treatment; (**B**) triglyceride levels in bovine mammary epithelial cells (BMECs) after treatment with the miR-143 mimic; (**C**) alteration in the number of lipid droplet in BMECs following miR-143 mimic treatment. ** *p* < 0.01.

**Figure 2:**
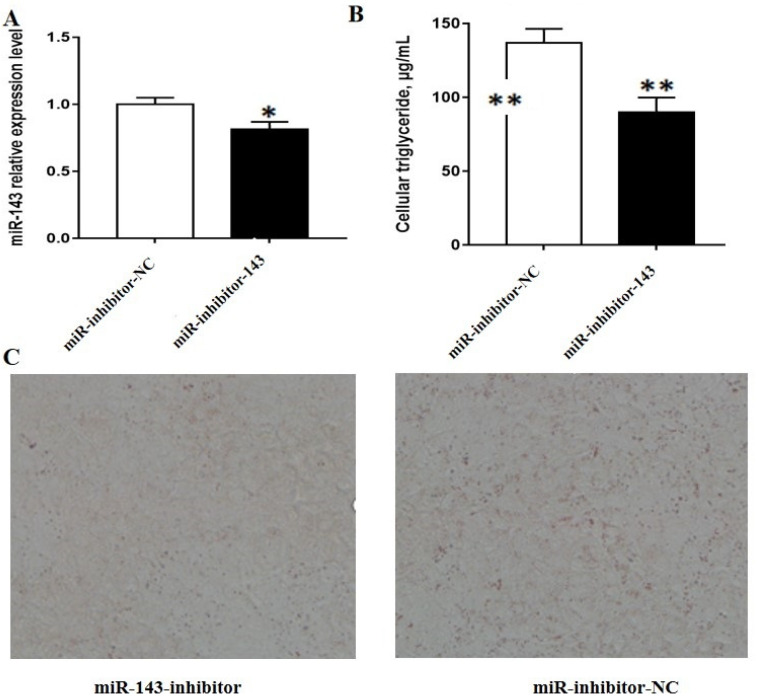
Effects of miR-143 inhibitor on milk fat synthesis. (**A**) miR-143 expression following miR-143 inhibitor treatment; (**B**) triglyceride levels in BMECs after treatment with the miR-143 inhibitor; (**C**) alterations in the number of lipid droplet in BMECs after miR-143 inhibitor treatment * *p* < 0.05; ** *p* < 0.01.

The efficiency of miRNA mimic and inhibitor transfection were confirmed by RT-PCR. The results indicated that miR-143 mimic markedly increased miR-143 expression (Figure 1A), whereas miR-143 inhibitor considerably inhibited miR-143 expression (Figure 2A). To reveal the miR-143 role in lipid metabolism, we examined the levels of triglyceride and the accumulation of lipid drop following the overexpression or silencing of miR-143. We found that miR-143 mimic significantly increased TAG levels relative to the control group (Figure 1B) and led to a marked rise in the number of lipid droplets (Figure 1C). On the contrary, the number of lipid droplets and the level of TAG decreased significantly when miR-143 was inhibited (Figure 2B,C). Collectively, our findings indicate that miR-143 has played a promotive role in milk fat synthesis.

### 3.2. MiR-143 Regulates Lipid Metabolism-Related Genes in BMECs

**Figure 3:**
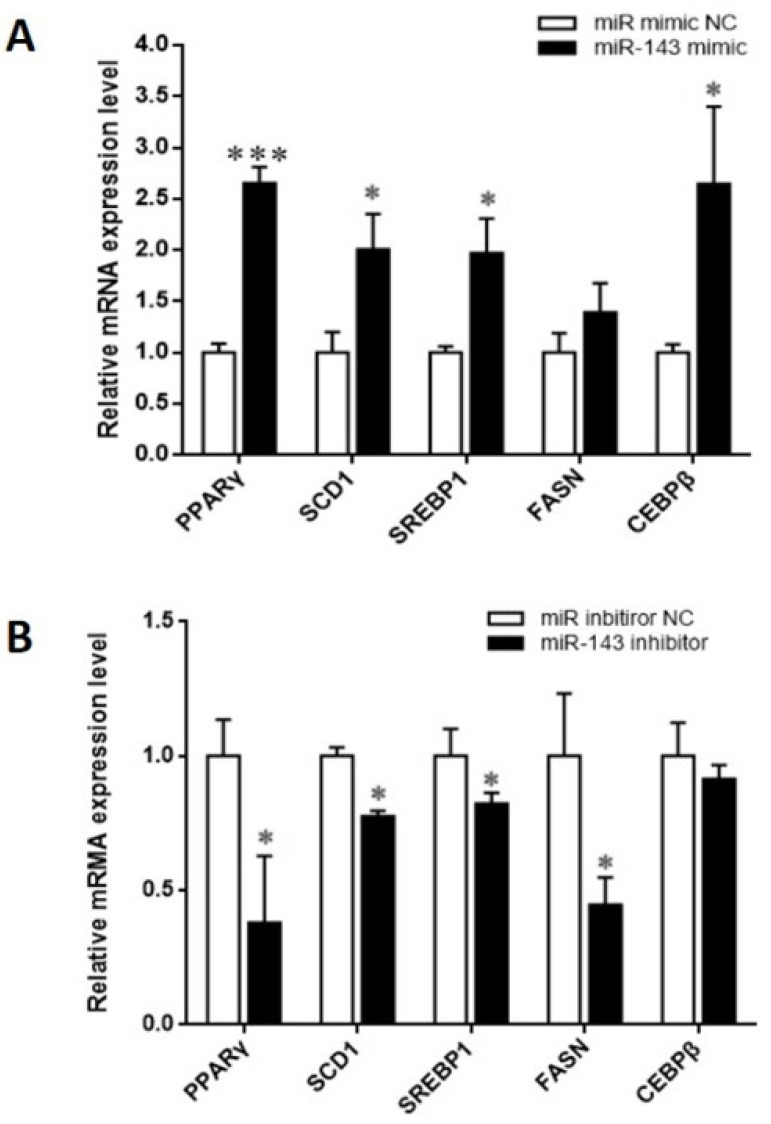
Effects of miR-143 on the expression of the genes related to lipid metabolism. (**A**) BMECs were transfected with miR-143 mimic; (**B**) BMECs were transfected with miR-143 inhibitor. * *p* < 0.05, and *** *p* < 0.001.

The genes previously identified to be associated with lipid metabolism were quantified via real-time PCR after transfection. miR-143 overexpression significantly upregulated lipid synthesis-associated genes such as PPARγ (*p* = 0.0001), SCD1 (*p* = 0.0120), SREBP1 (*p* = 0.008), CEBPβ (*p* = 0.0199), and FASN (*p* = 0.1199) (Figure 3A). However, we found that miR-143 inhibitor markedly down-regulated the expression of PPARγ (*p* = 0.0193), SREBP1 (*p* = 0.0463), FASN (*p* = 0.0193), and SCD1 (*p* = 0.0279) relative to the control group (Figure 3B).

### 3.3. Smad3 is a Target Gene of miR-143

**Figure 4:**
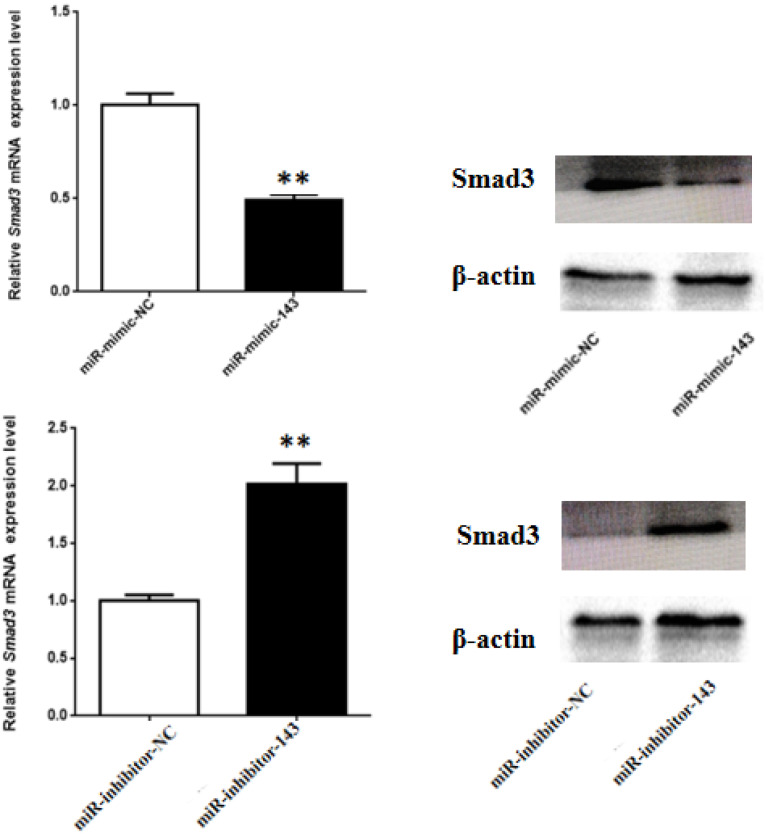
Impact of miR-143 on the Smad3 mRNA and protein expression. ** *p* < 0.01.

As Figure 3A shows, miR-143 mimic significantly down-regulated the Smad3 gene. In contrast, miR-143 inhibitor remarkably enhanced Smad3 gene expression (*p* < 0.01) (Figure 4). In parallel, Western blot assay revealed that the levels of Smad3 protein in miR-143 mimic and inhibitor transfection groups were the same as that of the mRNA expression response (Figure 4).

**Figure 5:**
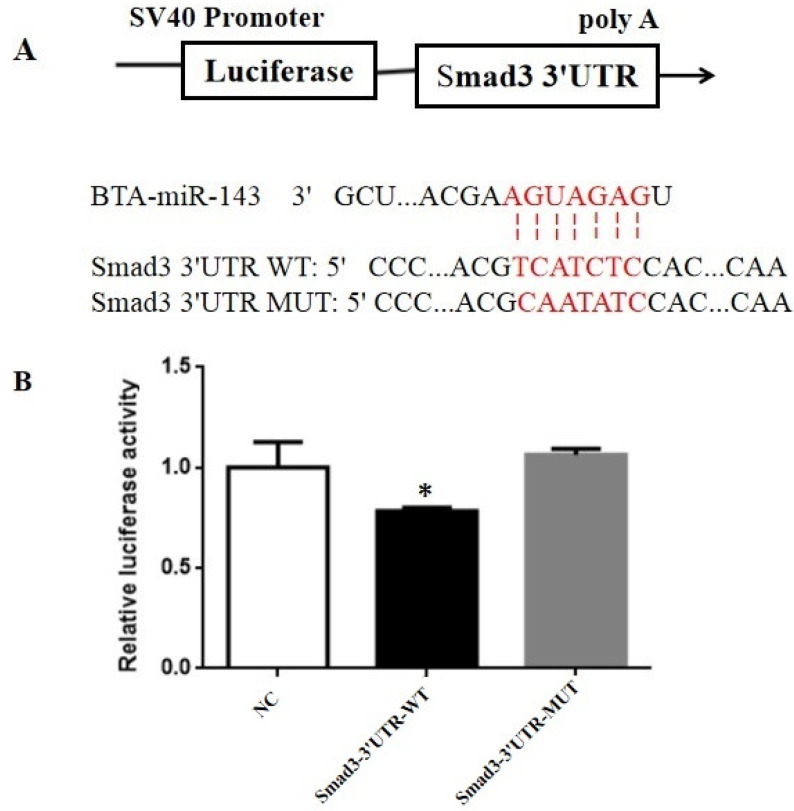
(**A**) Sequence alignment of miR-143 and the 3′-untranslated region (UTR) of Smad3 based on the TargetScan and miRDB algorithms; (**B**) changes in luciferase activity after the BMECs were co-transfected with miR-143 mimics and a luciferase reporter with a fragment of the Smad3 3′-UTR harboring either the miR-143 binding site (Smad3-3′UTR-WT) or a mutant (Smad3-3′UTR-MUT). * *p* < 0.05. SV40 = Simian virus 40; poly A = polyadenylic acid; BTA = *Bos taurus*.

In addition to the result projected by TargetScan, the Smad3 gene could be the miR-143 target gene (Figure 5A). So, the luciferase reporter system was employed to confirm whether the Smad3 gene was the target gene of miR-143. The result showed that the Smad3-3′UTR-WT group is significantly different from NC and Smad3-3′UTR-MUT (Figure 5B). Relative to the negative control group, miR-143 mimic substantially reduced the normalized luciferase activity by 23.4% (*p* = 0.042) (Figure 5B), whereas these repressions were completely abolished by the Smad-3′UTR MUT (Figure 5B).

### 3.4. siRNA-Smad3 Promotes Triglyceride Accumulation

**Figure 6:**
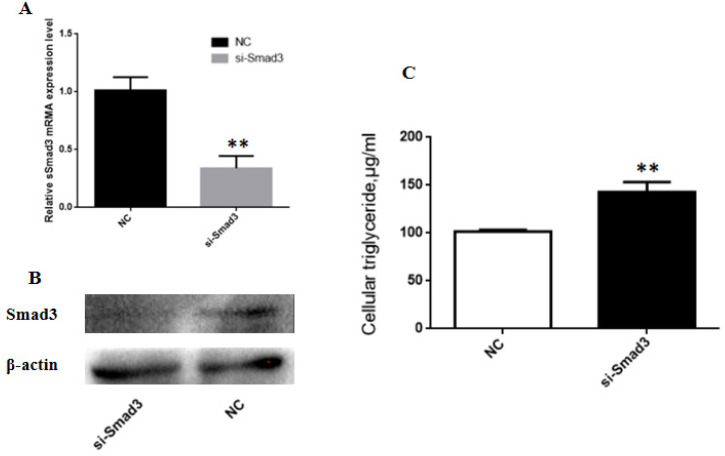
Effects of Smad3 on milk fat synthesis. (**A**,**B**) Comparative expression of Smad3 mRNA and protein in BMECs transfected with the si-Smad3 and negative control for 48 h. (**C**) Triglyceride levels in BMECs after treatment with the si-Smad3. ** *p* < 0.01.

To reveal the role of Smad3 on lipid metabolism in BMECs, Smad3 silencing assay was conducted with specific siRNA. The optimal siRNA concentration for transfection in BMECs was determined to be 100 nM and with high knock down efficiency (Figure 6A,B), and smad3 gene silencing significantly increased TAG level in BMECs (Figure 6C).

## 4. Discussion

Growing evidence has revealed that miRNAs play a crucial function in the milk fat synthesis [8,23,24,25]. miR-143 is one of the miRNAs closely related to lipid metabolism [14]; previous studies indicated that miR-143 participates in the differentiation of adipocytes, as well as triglyceride synthesis [15,16,17,18]. Furthermore, miR-143 was found to be differentially expressed in BMECs of high milk fat group and low milk fat group [6]. So, we centered on the function of miR-143 in milk fat synthesis. 

Herein, we found that miR-143 significantly promoted the triglyceride and lipid droplet accumulation in BMECs, which is consistent with previous work showing that miR-143 stimulates adipogenesis in mice and humans [18]. Furthermore, miR-143 increased the expression of PPARγ, SCD1, SREBP1, CEBPβ, and FASN. PPARγ, SREBP1, and C/EBPβ are key positive regulators of lipid accumulation and have been found to be significantly positively correlated with TAG content in BMECs and GMECs [26,27]. SCD1 is a lipogenic enzyme that regulates membrane lipid homeostasis and has been recognized as having large effects on milk fat composition [28]. FASN catalyzes fatty acid synthesis, and its expression is directly related to the occurrence of several milk traits, such as milk fat content and overall fatty acid composition [29]. Therefore, our results indicate that miR-143 might promote milk fat synthesis by affecting the expression of the key regulators of lipid accumulation in BMECs.

miRNAs can regulate multiple target genes, and exert their biological functions by different target genes in different cell types or in different cell states of the same cell type [30,31]. Chen et al. [32] and Kim et al. [33] revealed that miR-143 promotes adipogenesis by interacting with MAP2K5 and Pre-f-1 in adipocytes. Zhao et al. [34] found that miR-143 regulates the apoptosis and proliferative abilities of cervical cancer cells by interacting with HIF-1α. Herein, we verified that miR-143 targets Smad3 in BMEC using three different assays. Being an intracellular protein, Smad3 promotes TGF-β signal propagation to the nucleus from the cell membrane [35]. Yadav et al. [36] found that inhibition of Smad3 expression protects against obesity during high-fat feeding in vivo studies. Then in vitro experiments demonstrated that Smad3 mediates inhibition of adipocyte differentiation by TGF-β via inhibiting the expression of several adipocyte marker genes, including C/EBPs, PPARγ, and leptin [37,38,39]. Our results show that Smad3 gene silencing significantly increased the intracellular TAG level. So, we speculate that miR-143 promotes the formation of lipid droplet and TAG synthesis in BMECs via upregulation of the genes linked to lipid synthesis, like PPARγ, FASN, SCD1, CEBPβ, and SREBP1 by targeting Smad3.

## 5. Conclusions

Our results show that miR-143 participates in TAG synthesis in BMECs. Moreover, the results indicate that the miR-143–TGF-β/Smad3 axis might be vital for milk fat synthesis in BMECs. These results may assist in the development of unique strategies for improving beneficial milk components in dairy cows.

## Figures and Tables

**Table 1:** Sequence of primers for mRNA and miRNA quantitative real-time PCR.

Genes	Primer Sequence (5′-3′)	Annealing Temperature (°C)
UXT	F:TAGCCACCCTCAAGTATGTTCG	61 °C
	R:CGAGGTAGGAGGACAGGAGT	
PPARγ	F:AAAGGAGAGCCTGAACTTGGAG	61 °C
	R:TCTGAACTGTGCTGTGGCAA	
FASN	F:CCCTGAATGTGAGGCAGTGTG	61 °C
	R:TTAGCTGTGGTGAGGAGCCA	
CEBPβ	F:TGGTGAATAGTGCTGCCCAT	61 °C
	R:GGTGGTAGTTGTGGAAGCCC	
SCD1	F:ACATTGATCCCCACCTGCAA	61 °C
	R:AAACGTCATTCTGGAACGGC	
SREBP1	F:CAA TGTGTGAGAAGGCCAGT	61 °C
	R:ACAAGGAGCAGGTCACACAG	
Smad3	F: GAGTTGAAGCGAAGTTTGGGC	61 °C
	R: CTCTTGACTGCCTTCTCGCA	
U6	F:TAGCCACCCTCAAGTATGTTCG	61 °C
	R:CGAGGTAGGAGGACAGGAGT	
miR-143	GCTCGATGTCACGAAGTAGAGT	61 °C
